# The Hidden Flow Structure and Metric Space of Network Embedding Algorithms Based on Random Walks

**DOI:** 10.1038/s41598-017-12586-y

**Published:** 2017-10-13

**Authors:** Weiwei Gu, Li Gong, Xiaodan Lou, Jiang Zhang

**Affiliations:** School of Systems Science, Beijing Normal University, Beijing, 100875 P. R. China

## Abstract

Network embedding which encodes all vertices in a network as a set of numerical vectors in accordance with it’s local and global structures, has drawn widespread attention. Network embedding not only learns significant features of a network, such as the clustering and linking prediction but also learns the latent vector representation of the nodes which provides theoretical support for a variety of applications, such as visualization, link prediction, node classification, and recommendation. As the latest progress of the research, several algorithms based on random walks have been devised. Although those algorithms have drawn much attention for their high scores in learning efficiency and accuracy, there is still a lack of theoretical explanation, and the transparency of those algorithms has been doubted. Here, we propose an approach based on the open-flow network model to reveal the underlying flow structure and its hidden metric space of different random walk strategies on networks. We show that the essence of embedding based on random walks is the latent metric structure defined on the open-flow network. This not only deepens our understanding of random- walk-based embedding algorithms but also helps in finding new potential applications in network embedding.

## Introduction

Complex networks, as high-level abstractions of complex systems, have been widely applied in different areas, such as biology, sociology, economics and technology^1–6^. Recent progress has revealed a hidden geometric structure in networks^7,8^ that not only deepens our understanding of the multiscale nature and intrinsic heterogeneity of networks but also provides a useful tool to unravel the regularity of some dynamic processes on networks^7,9–14^. At the same time, researchers in the machine learning community have developed several techniques to embed a whole network in a high-dimensional space^15–20^ such that the vectors of each node can be used as abstract features feeding on neural networks to perform tasks. It has been demonstrated that such a form of network embedding has wide applications, such as community detection, node classification and link prediction^16,21^. Various methods have been proposed in network embedding field such as Principal Component Analysis, Multi-Dimensional Scaling, IsoMap and their extensions^22–26^. Those embedding methods give good performance when the network is small. But most of them cannot be effectively applied on networks containing millions of nodes and billions of edges.

Recently, there has been a surge of works proposing alternative ways to embed networks by training neural networks^15,16,27^ in various approaches inspired by natural language processing techniques^28–30^. To build a connection between language and network, a random walk needs to be implemented on the network such that the node sequences generated by random walks are treated as sentences in which nodes resemble words. After the sequences have been generated, skip-gram in word2vec^30^, which is one of the most famous algorithms for word embedding developed in the deep learning community, can be efficiently applied on the sequences. Among these random-walk-based approaches, deepwalk^15^ and node2vec^16^ have drawn wide attention for their high training speed and high classification accuracy. Both algorithms regard random walks as a paradigmatic dynamic process on a network that can reveal both the local and global network structures. Several extended works that unravel the fundamental co-occurrence matrix between the context and words in skip-gram-based embedding algorithms and the multiple-step transition matrix. Levy *et al*.^31^ proves that skip-gram models are implicitly factorizing a word-context matrix. Tang *et al*.^17^ takes 1-step and 2-step local relational co-occurrence into consideration, Cao *et al*.^18^ believes that the skip-gram is an equally weighted linear combination of k-step relational information. Those works were proposed soon after word2vec was presented. New progress includes the combination of random surfing strategy^15^, Levy *et al*. method^31^, and deep neural network^27^, and the consideration of asymmetric transitivity in directed network^32^.

Although random-walk-based embedding algorithms such as word2vec and node2vec are successfully applied in some real problems, several drawbacks still exist. First, explicit and fundamental explanations are needed to explain why neural-based algorithms work so well since these algorithms are fundamentally black boxes. Second, how to set the values of the hyper-parameters is still poorly understood. Third, explicit and intuitive explanations of the embedding vectors of each node and the inner structures of the embedding space are needed. We should find an explanation to provide a general framework to unify deepwalk, node2vec and other random walks based algorithms.

In this paper, we propose a new perspective based on a metric defined on the flow structures to understand the embedding space behind the random-walks-based algorithms, and accordingly we put forward a novel network embedding algorithm which combines the manifold learning with the new metric. First, we use the open-flow network model to characterize the overall structural and dynamical features of different random walk strategies on the same background network. Then, we note that there is a natural metric called the flow distance which is defined on these flow networks. Further, we discover that the hidden metric space framed by the flow distances is similar to the embedding space derived from the deepwalk and node2vec algorithms, since the euclidean distance derived from the embedding vectors are highly correlated with the flow distance. Finally, we propose a new network embedding method named Flow-based Geometric Embedding(FGE) with its numeric approximate improvement algorithm which has less free parameters and faster implementation than the known algorithms based on random walks. This embedding method can achieve similar clustering results with node2vec and reasonable ranking outcome compared with other ranking algorithms.

## Methods

Both deepwalk and node2vec are aim to learn the continuous feature representations of nodes by sampling truncated random walk sequences from the graph as mimic sentences to feed on the skip-gram^30^ which is an effective and efficient algorithm to learn word representations. The difference between node2vec and word2vec lies in the random walk strategy, the deepwalk algorithm implements a common unbiased random walk on a graph such that all the edges are visited in accordance with the relative weights on the local node, while node2vec employs a biased random walk in which the probability of visiting is adjusted by two parameters *p* and *q*. Node2vec can uncover much richer structures of a network because it resembles deepwalk when *p* = 1 and *q* = 1. Thus, we discuss only node2vec in the rest of this paper. Please refer to algorithms 4 and 5 to learn more concrete details about node2vec.

### Constructing Open-flow Networks

**Figure 1:**
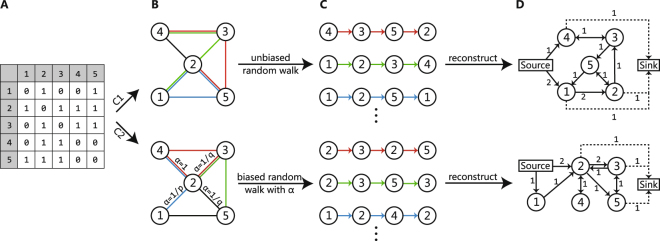
Illustration of the construction of different open-flow networks from the same background network with different random walk strategies. (**A**) represents the adjacency matrix of a network; (**B**) shows the random walks implemented by the deepwalk algorithm with *p* = 1, *q* = 1(C1), and node2vec algorithm with *p* = 0.5, *q* = 1 (C2) from (**A**); (**C**) shows several sequences of nodes generated by the corresponding random walk algorithms; (**D**) shows the open-flow networks constructed by the sequences. Algorithm 1 shows how to build an open-flow network matrix based on total flow from node to node.

To reveal the flow structure behind a random walk strategy (for a given *p* and *q*), we construct an open-flow network model^33^ in accordance with the random walk strategies. An open-flow network is a special directed weighted network in which the nodes are identical to those of the original network, and the weighted edges represent the actual fluxes realized by a large number of random walks. There are two special nodes, the source and the sink, representing the environment, that is why the network is called an open network. When a random walker is generated at a given node, a unit of flux is injected into the flow network from the source to the given node, and this particle contributes one unit of flux to all the edges visited. When the random walk is truncated, a unit of flux is added from the last node to the sink. A large number of random walkers jumping on the network according to the specific strategy form a flow structure that can be characterized by the open-flow network model, in which the weight on the edge $i\to j$ is the number of particles visited. Figure 1 illustrates how the different open-flow networks are constructed for a single background binary network with deepwalk in the upper panel and node2vec in the lower panel.

### Calculating Flow Distance

**Figure 2:**
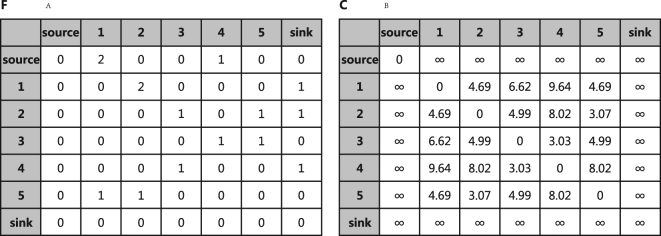
An example flow network including 7 nodes. (**A**) is the flux matrix *F* of the sampled network under condition C1 (*p* = 1, *q* = 1) in Fig. 1. (**B**) shows the flow distances among all nodes, where infinity means that there is no connected path from *i* to *j*. Algorithm 1 shows how to compute the flow distance based on the *F* matrix.

For a given flow network *F* with (*N* + 2) × (*N* + 2) entries, where the value at the *i*-th row and the *j*-th column represents the flux from *i* to *j*, the source is represented as the first node while the sink is represented as the last, the flow distance *c*_*ij*_ between any pair of nodes *i* and *j* is defined as the mean average number of steps needed for a random walker to jump from *i* to *j* for the first time and finally return back to *i* along all possible paths in the network^33^. It can be expressed as:

1 $${c}_{ij}=\frac{{(M{U}^{2})}_{ij}}{{u}_{ij}}+\frac{{(M{U}^{2})}_{ji}}{{u}_{ji}}-\frac{{(M{U}^{2})}_{ii}}{{u}_{ii}}-\frac{{(M{U}^{2})}_{jj}}{{u}_{jj}}$$

where, m_*ij*_ is the transition probability from *i* to *j*, which is defined as: ${m}_{ij}=\frac{{f}_{ij}}{{\sum }_{j\mathrm{=1}}^{N+1}{f}_{ij}}$ where *f*_*ij*_ is the total flow from node *i* to node *j*. The pseudo probability matrix *U* is defined as^33^:

2 $$U=I+M+{M}^{2}+\mathrm{...}\,=\,{(I-M)}^{-1}$$

where *I* is the identity matrix with *N* + 2 nodes. *u*_*ij*_ is the pseudo probability that a random walker jumps from *i* to *j* along all possible paths. Figure 2 is a sample flow network constructed under condition 1 in Fig. 1. Algorithm 1 shows the concrete details about how to calculate flow distance based on *F* matrix. However, when computing the flow distance we need to invert the matrix *I* − *M*, the complexity is O(|*N*|^3^) which is prohibitive for many large graph datasets and it is far slow than other recent embedding approaches. To conquer this problem, Ahmed *et al*.^34^ proposed a factorization technique so as to minimize the number of neighboring nodes. Here, we propose another method to compute the approximate value of flow distance which avoiding the time consuming calculation of matrix inversion. This new algorithm named Numerical Flow Distance Computing. It is much faster than the Analytical Flow Distance Computing (Equation 1). It’s time complexity is O(|*N*|^2^), where |*N*| is the number of nodes. The basic idea of this new algorithm is that the average flow distance between two nodes can be estimated directly from the node sequences generated by the random walk. We can just count how many nodes separating node *i* and node *j* in a given sampled node sequence generated by the random walk strategy, and then average them for a large number of sampled sequences to obtain the estimation of the flow distance between *i* and *j*. According to the large number theorem, this average value can approach the theoretical result calculated by Equation 1 if the number of sampling is sufficiently large. We apply the Numerical Flow Distance Computing and the Analytical one on Karate, Les Misérables and Airline networks. The correlation coefficient over those datasets is 0.96 in average, which indicates the numeric algorithm can obtain a good estimation of the analytical distance. The concrete details about the numerical computing method is listed in Algorithm 2.

### Embedding Networks

To display the hidden information in an open-flow network and visualize the node relationships, we embed nodes into a high-dimensional euclidean space according to flow distances (*c*_*ij*_). We use the SMACOF algorithm^35^ to perform the embedding. This algorithm takes the distance matrix and the number of embedding dimension as input, and tries to place each node in an *N*-dimensional space such that the between-node distance is preserved as well as possible. After this embedding process, we can achieve proper vector representation for each node. Please refer to algorithm 3 for more concrete details about this embedding method. The combination of flow distance 8, 9 and the manifold learning method 3 is named as Flow-based Geometric Embedding (FGE). In our paper, we apply the analytical method to compute the flow distance when the network’s node number is less than 1,000, we name this embedding as Analytical FGE. While the network has more than 1,000 nodes, we apply the numerical method and name this embedding as Numerical FGE. An overview of the networks used in our experiments is given in Table 1.

**Table 1:** An overview of the basic information of the datasets.

NAME	NODES	EDGES	DIRECTED
Les Misérables network	77	254	directed
Airline Network	3,425	67,333	directed
Karate Graph	34	156	undirected
Blog Catalog	10,312	333,983	undirected
Wikipedia	9488	832,408	directed weighted
China Click Websites	20,746	135,770	directed weighted

Les Misérables is a co-occurrence network with 77 characters and 254 relationships in the novel Les Misérables. Airline Network contains 59036 routes between 3209 airports on 531 airlines spanning the globe, the routes are directional. Karate Graph is a social network of friendships between 34 members of a karate club at a US university. Blog Catalog is a network of 333,983 social interactions of 10,312 bloggers listed on the Blog Catalogue website. Wikipedia datasets comes from the latest web page texts from the Chinese Wikipedia with 9488 nodes and 832,408 edges. China Click Websites contains 120 million records of all the clicking behaviours of 1000 users with 20,746 websites and 135,770 click streams within one month.

## Results

In this section, we present our results by applying FGE on several empirical networks and comparing with other embedding algorithms. Our main findings are included as follows.

- We notice that the open flow network model can be used to reflect the flow structure behind different random walk strategies;
- We discover that there is a high correlation between the flow distance and the euclidean distance calculated by the embedding results of node2vec algorithm for any node pair, therefore, the embedding results of FGE and node2vec are highly correlated compared to other known embedding algorithms;
- We infer that there is a hidden metric structure in the embedding vector space, and this metric structure can be used for clustering and ranking nodes.

### Flow Structure and Representation

**Figure 3:**
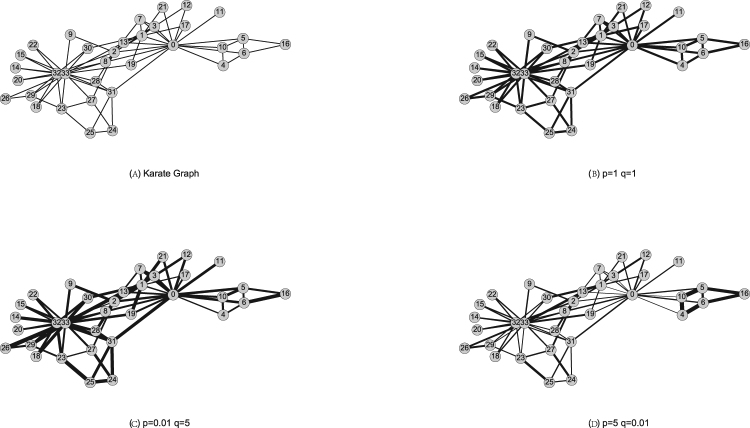
Visualization of flow network structure. (**A**) is an undirected, unweighted network of Karate Graph. (**B**) is the result of unbaised random walks on this graph. (**C**) indic ates the random walks mainly explored within a community to uncover the local structures, while in (**D**) the vast flows between different communities showed the random walks were trying to find the global structures in this network.

**Figure 4:**
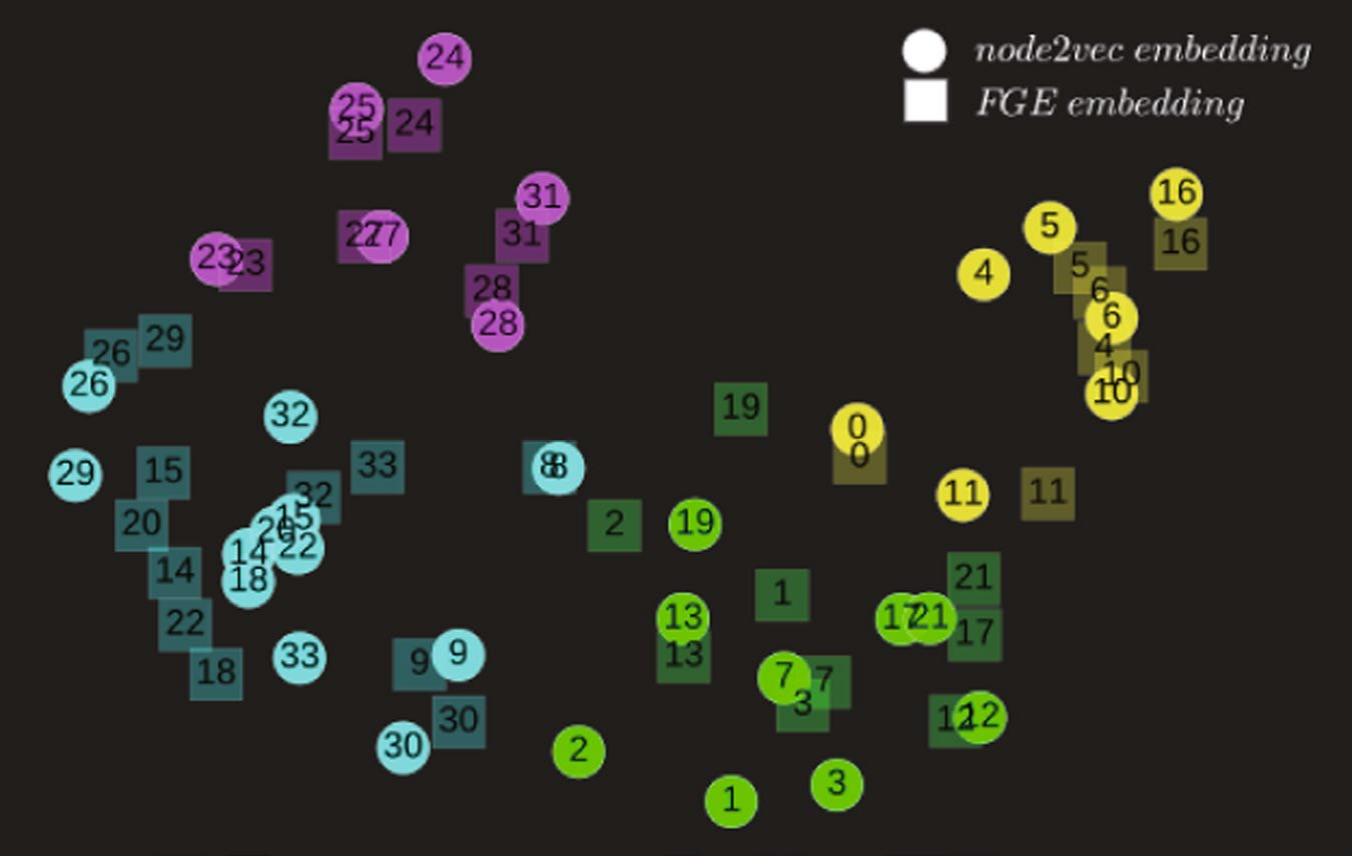
The embedding of Karate Graph. The visualization results were generated by node2vec and FGE algorithms with label colors reflecting clustering results and node shapes indicating different embedding methods.

To demonstrate our first finding, we use Karate Graph, a small but representative network, as our experimental ground. Figure 3 shows different flow structures of node2vec algorithm under different *p* and *q*, where the thickness of edges indicates the amount of flows between nodes. To capture the hidden metric on the flow structures, we fed random walk sequences into node2vec and FGE algorithms with the number of walks per node *r* = 1024, walk length *l* = 10, and embedding dimension *d* = 64. After training process, each node acquires two vector representations, denoted by *θ* in FGE and *π* in node2vec. We then visualize the vector representations using t-SNE^36^, which provides both qualitative and quantitative results. Figure 4 visualize the flow structure generated by un-biased random walk strategies under *p* = 1, *q* = 1. Intuitively, we observe that the nodes represented by node2vec embedding and FGE embedding almost overlapped each other. This indicates that the flow distances in FGE algorithm captures the essence of node2vec. Additionally, the latent relationship between nodes is well expressed. For example, we find that nodes 4, 5, 10 and 16 are all close to each other and they belong to the same community in both algorithms. By analyzing the network structure, we also discover that nodes 14, 15, 20, and 22 are much closer to each other in node2vec embedding than in the FGE. That may due to that node2vec only considers n-steps connection between nodes. However, this difference can be captured by FGE algorithm since it considered all pathways and infinity steps’ relations.

### Correlations between Distances

**Figure 5:**
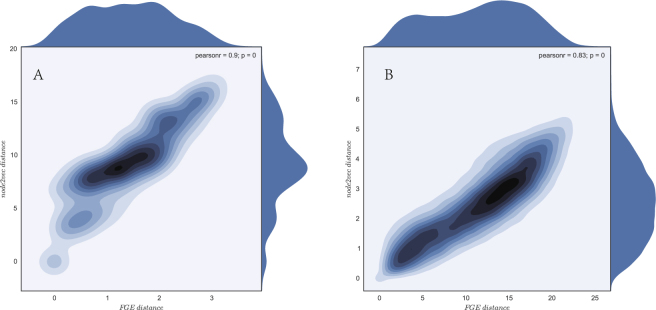
Heat maps of flow distance and node2vec distance under p = 1, q = 1. (**A**) shows the correlation coefficient between flow distance and node2vec distance of Karate Graph. (**B**) indicates the correlation coefficient of Airline Network dataset.

To confirm our conclusion that there is a hidden metric space behind the random-walk-based network embedding algorithms, we plotted the flow distance of FGE and the euclidean distance of node2vec embedding on the same network. Both algorithms based on the same node sequences and background network. The results show that flow distance and node2vec’s euclidean distance are highly correlated. Figure 5 is a heat map, where the X-axis represents the flow distance between nodes *i* and *j*, and the Y-axis denotes nodes’ node2vec distance. The Pearson correlation between the two distances is 0.90 with a p-value 0.001 in Fig. 5A and 0.83 with a p-value = 0 in Fig. 5B. The correlation indicates there is a highly linear relationship between the paired nodes in FGE and node2vec.

To demonstrate the generality of our second finding that the flow distance is highly correlated with the node2ve’s euclidean distance. We compare the euclidean distances of nodes’ vector representations derived from node2vec with several baseline algorithms such as LINE^17^, Spectral Clustering^37^, PPMI^31^ and Jaccard overlap We perform the same experiments on several different datasets. Table 2 shows that there is a high correlation between the flow distance and the node2vec’s distance and that correlation is not sensitive to the walking strategies(different *p* and *q*). This may due to that different walking strategies can generate different neighbor nodes which can be captured by different open flow network models. Thus the flow distance can reveal the latent space in random-walk-based network embedding algorithms. Figure S1 in Supplementary material shows the correlation values between node2vec’s distance and the euclidean metric of all other baseline algorithms. The highest correlation is highlighted in bold for each row. Overall, the flow distance’s correlation is significantly higher than others. In the supplementary material, a rough mathematical illustration is provided which explains why the correlation is high between flow distance and euclidean distance in node2vec embedding space.

**Table 2:** The correlation coefficients of different datasets.

Parameter	Karate Graph	Les Misérables Network	Airline Network	Wikipedia
p = 1.0, q = 1.0	0.91	0.92	0.81	0.72
p = 0.5, q = 2.0	0.90	0.91	0.83	0.70
p = 2.0, q = 0.5	0.91	0.91	0.81	0.71

This table shows the coefficients between flow distance and node2vec’s euclidean distance in embedding space, in which node2vec is chosen with different “inward” and “outward” parameters (*p* and *q*).

### Node Clustering

To further prove the similarity between FGE and node2vec, we compare their performance in node clustering task. In complex network studies, node clustering is a vital task in community structure detection, which is of importance in various backgrounds^38–40^. We perform the k-means clustering on the node vectors *θ* and *π* acquired from FGE and node2vec with the same random walk sequences. In other words, we control walk times, walk length and embedding dimensions for both algorithms. The average silhouette coefficient is used to determine the number of clusters. Here, we visualize the clustering results on the Karate network in Fig. 4. According to the silhouette value, the Karate graph can be divided into 4 clusters, with each node’s color represents it’s community. As shown in Fig. 4, FGE and node2vec give identical cluster results on the Karate graph with number of walks *r* = 1024, embedding dimension *d* = 64 and walk length *l* = 10. To assess the quality of the clustering results, we reported the averaged Normalized Mutual Information(NMI) score^41^ over several baseline methods such as LINE^17^, Spectral Clustering^37^, PPMI^31^ and Jaccard overlap. As shown in Figure S2 in Supplementary material, the FGE method consistently has a higher nmi value compared with other baseline methods. For LINE and Spectral Clustering, increasing the embedding dimension does not appear to be effective in improving the nmi value.

### Centrality Measurement

Understanding the hidden geometry of a random walk strategy could provide new insights as well as new applications. We expand our applications of network embedding and the hidden metric to centrality measurement which is another key application in network analysis^42–44^. Through calculating flow distances between nodes, we can get reasonable ranking results by averaging nodes’ total flow distances. Formally, we define the centrality based on flow distances as:

3 $${\bar{c}}_{i}=\sum _{j}{c}_{ij}$$

where c_*ij*_ denotes the flow distance between node *i* and *j*, and the nodes with lower ${\bar{c}}_{i}$ values are more central than others. The reason for the usefulness of this definition is that the nodes close to others always have tight connections and high traffics. Since the flow distance is highly correlated with node2vec’s euclidean metric, thus this definition also works in node2vec embedding. We can measure each node’s centrality through its distance to all other nodes in the embedding euclidean space. After that, we can read the centrality information directly from the visualization of the embedded graph because the nodes with high centrality (small average distances to others) are always concentrated on the central area on the visualization.

We applied node2vec and flow distance’s centrality measuring algorithms on China Click Websites, which contained approximately 5 years of browsing data from more than 30000 online volunteers. We calculated each website’s centrality based on its flow distances and node2vec’s euclidean distances. We discovered that the most popular websites always have small distance because they usually have more traveling paths to other websites. Therefore, the smaller the distance, the more central the website position is. We ranked the websites in accordance with their centrality and then compared flow distance and node2vec’s euclidean distance with PageRank and total traffic (the number of clicks for each website). The ranking results for the top 10 websites are listed in Table 3. We find that the ranking orders of the flow distance and node2vec are nearly the same. We also discover that the high-traffic websites, such as Tmall.com (a popular shopping website) and 163.com (a popular mailbox website) have lower ranks, but baidu.com and qq.com have higher ranks even though their total traffics are not heavy. That is because baidu.com and qq.com are bridges between the real and virtual worlds.

**Table 3:** Centrality ranking of top 10 websites.

rank	web name	flow distance	node2vec distacne	PageRank	total flow
1	baidu	(1)26.332	(1)26.437	(1)0.0221	(1)105560
2	qq	(2)30.087	(2)30.208	(2)0.0189	(2)57209
3	sogou	(3)33.035	(4)33.168	(3)0.0131	(4)25979
4	taobao	(4)33.272	(3)33.106	(4)0.0120	(3)35311
5	hao123	(5)33.626	(5)34.190	(6)0.0122	(5)23295
6	sina	(6)33.818	(7)33.954	(5)0.0098	(7)21711
7	weibo	(7)34.054	(6)33.953	(9)0.0070	(6)21815
8	163.com	(8)34.949	(12)36.41	(7)0.0062	(12)13890
9	sohu	(9)35.706	(8)33.239	(8)0.0071	(8)15512
10	360	(10)35.01	(9)35.155	(10)0.006	(9)14711

Ranking top 10 websites according to flow distance, node2vec distance and comparisons with other ranking methods.

### Parameter Sensitivity

**Figure 6:**
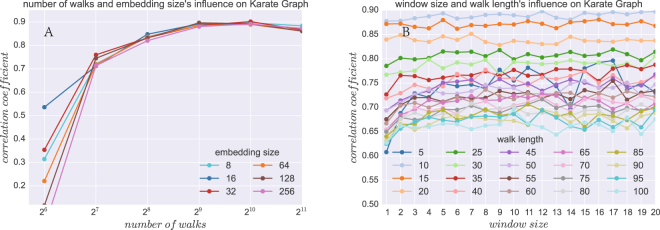
Parameter Sensitivity Study. (**A**) The correlation coefficient over embedding size *d* and number of walks per node *r*. (**B**) The correlation coefficient over walk length *l* and number of walks per node *r*.

Random-walk-based embedding algorithms involve a number of sensitive parameters. To evaluate how those parameters affect the correlation between flow distance and node2vec’s euclidean distance, we conduct several experiments on the Karate graph. We examine how the embedding dimension *d*, the number of walks *r*, the window size *w*, and the walk length *l* influence the correlation between two distances. As shown in Fig. 6A, the correlation grows as the number of walks increasing, the correlation tends to saturate when *r* reaches 512. (Fig. 6A). However, there is a slight trend of correlation decreasing as the number of walks increasing. We speculate that this may due to errors in the substitution of the large sample of random walks using the open-flow network. The FGE algorithm assumes that the random walks can be represented as a Markovian process on the network, which means that each step jump is exclusively determined by the previous-step position. However, the random walk of node2vec does not satisfy this condition. Even though the difference exists as seen in Fig. 6B. We believe that the hidden metric of flows is more essential to reflect the structural properties of the network. We also evaluate how changes to the window size *w* and walk length *l* affected the correlation. We have fixed the embedding size and the number of walks to sensible values *d* = 128, *r* = 512 and then begin to vary the window size *w* for node2vec embedding and walk length *l* for random walks. The correlation variances are not that large when *w* changes. When the walk length *l* reached 10, the correlation declines rapidly with further increases in the walk length. We have done parameter sensitivity studies on other datasets, and discovered that the walk length parameter has a great influence on correlation values. When the network become larger, the walk length need to be increased to meet the high standard of correlation.

### Scalability

**Figure 7:**
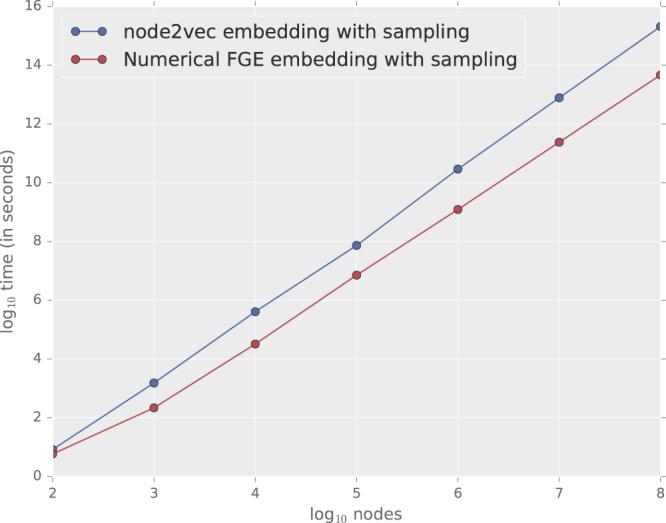
Scalability of Numerical FGE and node2vec on Erdos-Renyi graphs with an average degree of 10.

To test scalability, we obtain node representations by using node2vec and the Numerical FGE embedding with default parameter values for Erdos-Renyi graphs with increasing network sizes from 100 to 100,000,000 nodes and constant average degree of 10. In Fig. 7, we empirically observe that both node2vec and FGE scale linearly with increase in the number of nodes. The representations for 100 million nodes can be generated in less than several hours. The optimization phase is efficient by using negative sampling and asynchronous SGD. This figure shows that the Numerical FGE algorithm is consistently faster than node2vec especially for networks with millions of nodes.

**Algorithm 1:**
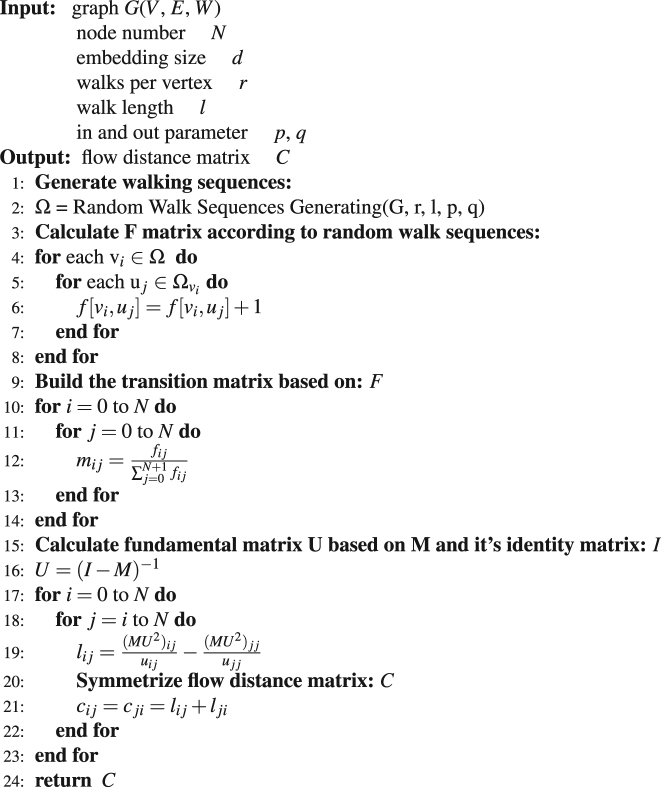
Analytical Flow Distance Computing (*G*, *d*, *r*, *l*, *p*, *q*),*N*.

**Algorithm 2:**
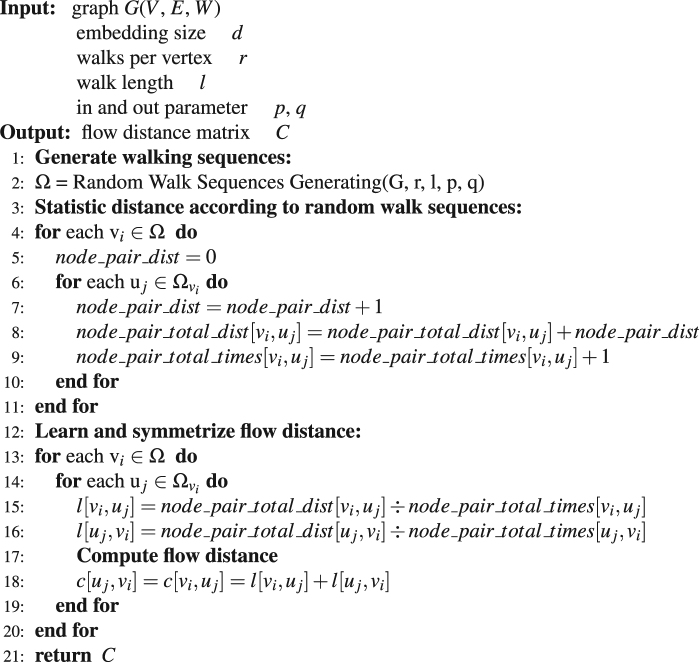
Numerical Flow Distance Computing (*G*, *d*, *r*, *l*, *p*, *q*).

**Algorithm 3:**
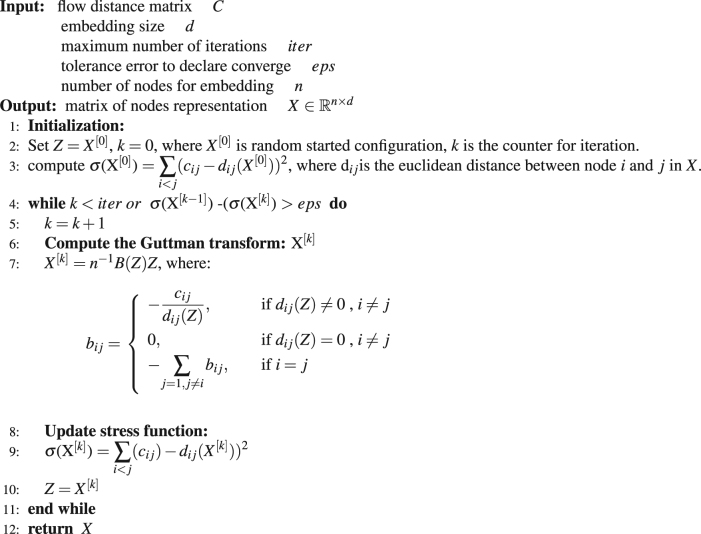
Network Embedding (*C*, *d*, *iter*, *eps*, *n*).

**Algorithm 4:**
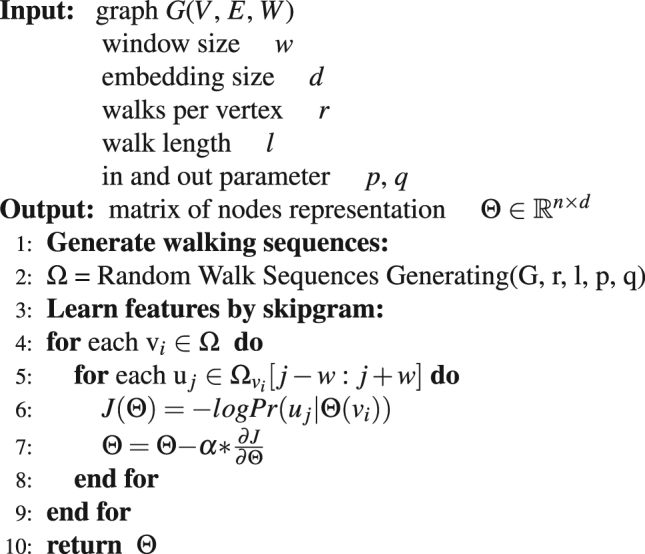
Node2vec Embedding (*G*, *w*, *d*, *r*, *l*, *p*, *q*).

**Algorithm 5:**
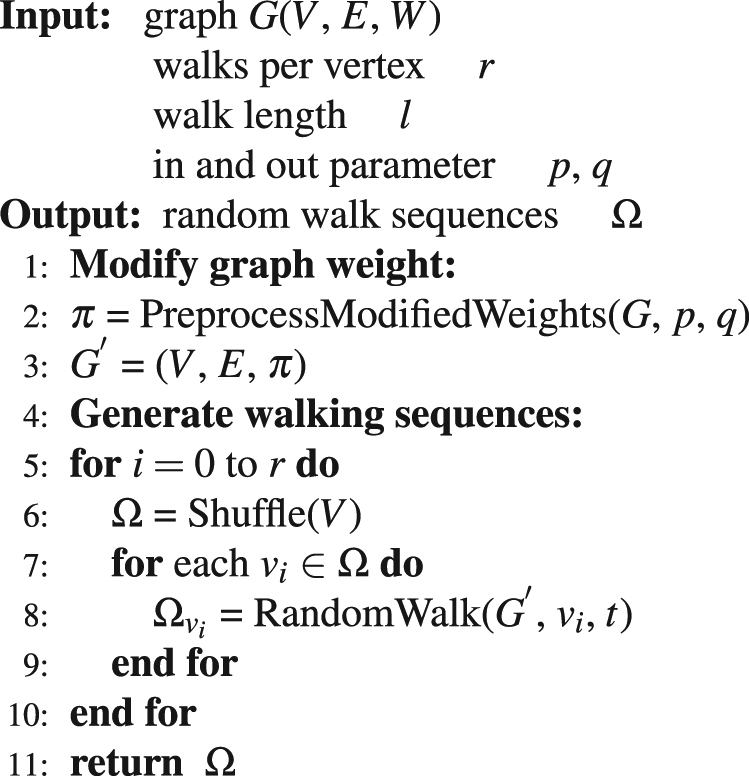
Random Walk Sequences Generating (*G*, *r*, *l*, *p*, *q*).

## Conclusions and Discussions

In this paper, we revealed the hidden flow structure and metric space of random-walk-based network embedding algorithms by introducing flow distance as well as FGE algorithm. The FGE algorithm learns nodes representation that encoding both structural and local regularities. The high Pearson correlation value between the node2vec representations and FGE vectors indicates that there is a hidden metric of random-walk-based network-embedding algorithms. The FGE algorithm not only helps in finding the hidden metric space but also works as a novel approach to learn the latent relations between vertices. Experiments on a variety of different networks and baseline methods illustrated the effectiveness of this embedding method in revealing the hidden metric space of random-walk-based network embedding algorithms. This finding not only provides a novel perspective to understand the essence of network embedding based on random walks but also reveals the hidden metric euclidean space behind those random-walk based embedding algorithms. With this understanding, we first applied node2vec to centrality measuring task. We also validated the functional correlation between FGE and node2vec in clustering task. The outcome showed that the two algorithms give similar clustering results, the NMI value is much higher compared with other baseline algorithms. The FGE algorithm has less free parameters, and the Numerical FGE method is much faster than node2vec. PPMI^31^ proved that the skip-gram in word2vec is implicitly factorizes a word-context matrix. In the future, we would like to explore the hidden relationship between the flow distance and the point wise mutual information. Both node2vec and FGE regard random walk as a paradigmatic dynamic process to reveal network structures. This sampling strategy consumes a large amount of computer resources to reach a stationary state for each node. Further extensions of FGE could involve calculating the nodes’ flow distances without sampling.

### Dataset and code

We have published our data and code to support this manuscript. Here is the link to access our code and dataset: https://github.com/Villafly/FGE_algorithm.

## Electronic supplementary material

Supplementary information
