# Supplementary material for: The Hidden Flow Structure and Metric Space of Network Embedding Algorithms Based on Random Walks
Source: Sci Rep. 2017 Oct 13;7:13114. doi: 10.1038/s41598-017-12586-y (PMC5640610; doi:10.1038/s41598-017-12586-y)
Supplement: Supplementary file 1 — Supplementary information [file 41598_2017_12586_MOESM1_ESM.pdf]

Supplementary Information

The Hidden Flow Structure and Metric Space of Network Embedding Algorithms Based on Random Walks

Weiwei Gu ,Li Gong, Xiaodan Lou, Jiang Zhang*

1 Rationale behind the high correlation between FGE and random walk based algorithms

In this paper, we present an novel node embedding algorithm, named Flow-based Geometric Embedding (FGE), which combines a manifold learning algorithm with a flow-distance metric. We discuss connections between this algorithm and previous works, such as node2vec, and show that the node2vec's distance metric is highly correlated with the flow-distance. This section provides a roughly description of the rationale behind the correlation. Some of the information given in the main body of the article is reproduced here to make this supplement self-contained.

Roughly speaking, the optimization process of skip-gram is equivalent to adjusting the vectors of nodes to reflect the flow distances of random walk. According to the log-likelihood of skip-gram (the log-likelihood of negative samples is ignored to facilitate our discussion):

$$L = \sum_{w}\sum_{c} \#(w, c)\log(\sigma(v_w \cdot v_c)) \tag{1}$$

We know that if the pair of (w, c) always co-occur in the sequences generated by the random walk, then the vector representations of w and c should be similar, otherwise, the vector representations of w and c should be dissimilar. Note that all the sequences are generated by the random walk on the original network, therefore, if two nodes i and j are close each other, they should have shorter flow distance as well as higher probability for co-occurring in a context of random walk according to the Markovian dynamics. In this way, shorter flow distance implies larger co-occurrence probability. Mathematically we know that,

$$\sum_{c} \#(i, c) = \sum_{j}\sum_{q<s}\sum_{c(j,q)} \#(i, c(j, q)) \approx \sum_{j}\sum_{c(j,t)} \#(i, c(j, t)) \tag{2}$$

where $c(j, q)$ represents the context that node j appears in the qth location, and s is the window size, t is the average distance from i to j for all q and $c(j, q)$, it is the flow distance between i and j. And we know that:

$$\#(i, c(j, t)) = NP(i, c(j, t)) = NP(i)P(x_1|i)P(x_2|x_1)...P(j|x_{t-1}) \tag{3}$$

where, N is the total times of random walks, x_1, x_2, , x_{t-1} are the nodes of the 1st, 2nd ,?. , (t ? 1)th nodes in the path $c(j, t)$. We denote $P(i)$ as m_0, and $P(\mathrm{x}_{k+1}$—$\mathrm{x}_k)$ as m_k, thus, we have:

$$\#(I, c(j, t)) = N\Pi_k^t m_k \tag{4}$$

And we know m_k varies in a certain statistical law, we treat them as i.i.d. random variables. We assume that the mean value of ?log m_k is ?, which must be a positive number, then when t becomes large and according to the large number theorem, we have:

$$\#(I, c(j, t)) = N\exp(\sum_{k} log(m_k)) \to N\exp(-t\mu) \tag{5}$$

*zhangjiang@bnu.edu.cn

Table S1. Comparing the correlation between node2vec's distance and other metrics

Dataset	Algorithm				
	Flow distance	Spectral Clustering distance	Jaccard distance	LINE distance	PPMI distance
Karate	**0.91**	0.79	0.76	0.56	0.78
Lesmis	**0.90**	0.62	0.45	0.42	0.79
Airline	**0.81**	0.35	0.77	0.30	0.62
Wiki	**0.82**	0.50	0.50	0.43	0.70
BlogCatalog	**0.79**	0.55	0.65	0.46	0.81

Table S2. Comparing the nmi value between node2vec's embedding with baseline methods

Dataset	Algorithm				
	FGE	Spectral Clustering	Jaccard embedding	LINE	PPMI
Karate	**0.91**	0.68	0.53	0.40	0.79
Lesmis	**0.92**	0.60	0.45	0.47	0.81
Airline	**0.87**	0.54	0.42	0.41	0.84

Because N is a constant, $\#(I, c(j,t))$ decays with t, the number of steps from i to j in the context c, that is the flow distance between i and j. To maximize the likelihood, we must maximize each component of L, that is

$$\#(i, c(j,t)) \log(\sigma(v_i \cdot v_{(j,t)})) \tag{6}$$

Therefore if i and j are close each other (t is small), their similarity $v_i \cdot v_{c_{(j,t)}}$ should be maximized, this imposes v_j being close to v_i because $v_{c_{(j,t)}}$ is the superposition of all the vectors in context $c_{(j,t)}$. Hence, larger flow distance t indicates dissimilar vector representations of i and j.

Although the distance between flow and node2ve is not total positive linear. The node2vec algorithm also affected by many factors, such as learning rate, window size and iteration times. Flow distance captures an underlying metric of node2vec embedding. With this understanding, we can expand node2vec's applications from classification, link prediction, clustering and visualization to centrality measuring. Through the hidden metric between nodes, we can get reasonable ranking results according to this metric.

2 Supplementary tables

Here, we give the empirical support for the main claim that the flow distance is highly correlated with the node2vec distance, there is a hidden metric space under the node2vec's embedding algorithm. We introduced the Pearson correlation coefficient and the Normalized Mutual Information to judge the distance similarity and the clustering consistency separately. In Table S1. we show the correlation values between node2vec's Euclidean distance and other baseline metrics over different datasets. In Table S2. we represent the nmi scores to judge the clustering results based on nodes vector representation. In order to get vector representation we embed the Jaccard distances into 2 dimension with Multi Dimension Scaling algorithm.
